# Letter to the Editor: Dopamine D2S/D2L Receptor Regulation of Alcohol‐Induced Reward and Signalling

**DOI:** 10.1111/adb.70117

**Published:** 2026-01-08

**Authors:** Mohsin Tariq, Muhammad Ahmad, Mian Zain Hayat, Meer Hassan Khalid

**Affiliations:** ^1^ Shaikh Khalifa Bin Zayed Al‐Nahyan Medical and Dental College Lahore Pakistan

Dear Editor,

I would like to commend Tayyab et al. [1] for their innovative and mechanistically rich investigation into the differential roles of D2S and D2L dopamine receptor isoforms in alcohol‐induced reward and intracellular signalling. Their work provides an important contribution to addiction neuroscience by dissecting isoform‐specific pathways that have historically been difficult to resolve using conventional pharmacological approaches. The study's integration of behavioural, molecular and receptor‐specific knockout strategies offers valuable insights into how distinct D2 receptor subtypes contribute to alcohol‐associated reinforcement. However, certain methodological and interpretative aspects warrant further discussion to strengthen the translational significance of these findings.

First, although the authors elegantly demonstrate isoform‐specific effects, the behavioural paradigms used including conditioned place preference (CPP) are susceptible to variability in motivational salience among rodents. For instance, prior work by Cunningham et al. [2] highlighted how environmental and handling factors significantly modulate CPP outcomes, suggesting that additional behavioural assays such as operant self‐administration may have enhanced robustness and reproducibility.

Second, the study's signalling analyses focus primarily on Akt/GSK3β and ERK pathways. While these are well‐established downstream targets of D2 receptors, they do not fully capture the complex signalling landscape implicated in alcohol reward. For instance, Beaulieu et al. [3] demonstrated that D2 receptor signalling involves β‐arrestin–dependent cascades that may diverge between D2S and D2L isoforms. Inclusion of such pathways would enhance mechanistic depth and clarify potential isoform‐specific signalling bifurcations.

Third, although the authors utilized D2S‐ and D2L‐specific knockout mice, developmental compensation remains a potential confound. For instance, as noted by Kelly et al. [4], constitutive deletion of dopamine receptor variants can lead to homeostatic rewiring of dopaminergic circuitry, potentially obscuring acute isoform contributions. Conditional or inducible knockout models could help mitigate these confounds and improve mechanistic precision.

Lastly, while the study compellingly links D2S to enhanced alcohol reward, long‐term neuroadaptive changes were not explored. For instance, Koob and Volkow [5] emphasized that addiction is driven by progressive transitions in reward, stress and executive control circuits. Longitudinal or chronic‐exposure models would therefore help determine whether D2 isoform‐specific signalling contributes to long‐term vulnerability rather than acute reward alone.

Future investigations should consider integrating multi‐modal behavioural paradigms, β‐arrestin pathway analyses, inducible genetic models and long‐term neuroadaptation studies to expand upon these promising findings. Such approaches would provide a more comprehensive understanding of how D2 receptor isoforms shape alcohol‐related reinforcement and potential therapeutic targets.

In conclusion, the work of Tayyab et al. [1] offers an important advancement in delineating the receptor‐level mechanisms underlying alcohol reward. Strengthening future research through complementary behavioural, signalling and longitudinal methodologies will be essential to fully appreciate the therapeutic relevance of D2S/D2L‐specific modulation in alcohol‐use disorders.

## Author Contributions

All the authors meet the ICMJE authorship criteria and have made significant and equal contributions to this manuscript. All authors approved the final version and agree to be accountable for all aspects of the work, ensuring the accuracy and integrity of the data and interpretation.

## Funding

The authors have nothing to report.

## Disclosure

All authors have read and approved the final version of the manuscript. They take complete responsibility for the integrity of the data and the accuracy of the data analysis. The authors affirm that this manuscript is an honest, accurate and transparent account of the work being reported, that no important aspects have been omitted and that any discrepancies from the planned study (and, if relevant, registered protocols) have been explained.

## Ethics Statement

The authors have nothing to report.

## Conflicts of Interest

The authors declare no conflicts of interest.

## Data Availability

Data sharing does not apply to this article as no datasets were generated during the current study; all data were sourced from published literature.
